# Using click chemistry to study microbial ecology and evolution

**DOI:** 10.1038/s43705-022-00205-5

**Published:** 2023-01-31

**Authors:** Sander van Kasteren, Daniel E. Rozen

**Affiliations:** 1 Leiden Institute of Chemistry and The Institute of Chemical Immunology, Leiden University, Einsteinweg 55, Leiden, 2300 RA The Netherlands; 2 Institute of Biology, Leiden University, Sylviusweg 72, Leiden, 2300 RA The Netherlands

**Keywords:** Microbial ecology, Microbial ecology

## Abstract

Technological advances have largely driven the revolution in our understanding of the structure and function of microbial communities. Culturing, long the primary tool to probe microbial life, was supplanted by sequencing and other -omics approaches, which allowed detailed quantitative insights into species composition, metabolic potential, transcriptional activity, secretory responses and more. Although the ability to characterize “who’s there” has never been easier or cheaper, it remains technically challenging and expensive to understand what the diverse species and strains that comprise microbial communities are doing in situ, and how these behaviors change through time. Our aim in this brief review is to introduce a developing toolkit based on click chemistry that can accelerate and reduce the expense of functional analyses of the ecology and evolution of microbial communities. After first outlining the history of technological development in this field, we will discuss key applications to date using diverse labels, including BONCAT, and then end with a selective (biased) view of areas where click-chemistry and BONCAT-based approaches stand to have a significant impact on our understanding of microbial communities.

## What is biorthogonal chemistry?

**Fig. 1:**
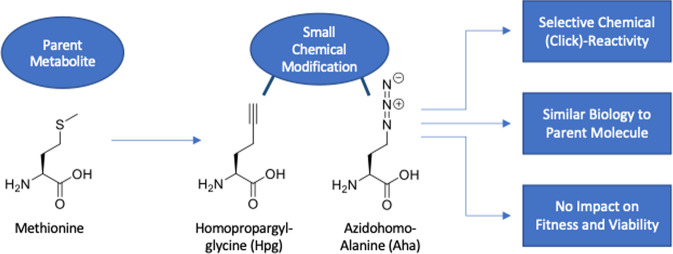
Overview of the metabolic labeling approach: a parent molecule (in this case methionine) is chemically altered in such a way that a selectively reactive chemical group is introduced whilst only minimally altering the biological function. In case of methionine (1), its reactive analogues Hpg (2) and Aha (3) are still transported into cells and incorporated by the tRNA/synthase pairs into nascent proteins.

Bioorthogonal chemistry, also known as click chemistry, is an approach in which a biomolecule is labelled with a small chemical group that can be subsequently ligated to a detectable group. In practice, this means the following: (1) a small molecule, like a cell-wall component, an amino acid, a nucleotide, or a metabolite, is modified with a chemical “handle” that does not affect its biological function (the molecule is therefore “bioorthogonal”); (2) this “handle” is then attached to a second molecule containing e.g., a fluorescent tag, which allows any process in a cell that uses the original molecule to be visualized, quantified, or selectively isolated (exemplified by methionine (**1**) and derivatives (**2** and **3**) in Fig. 1). As we describe below, this flexible approach is powerful because it labels and differentiates cells on the basis of their function or their behavior, especially when used in conjunction with other existing -omics methods.

## A brief history of bioorthogonal chemistry

**Fig. 2:**
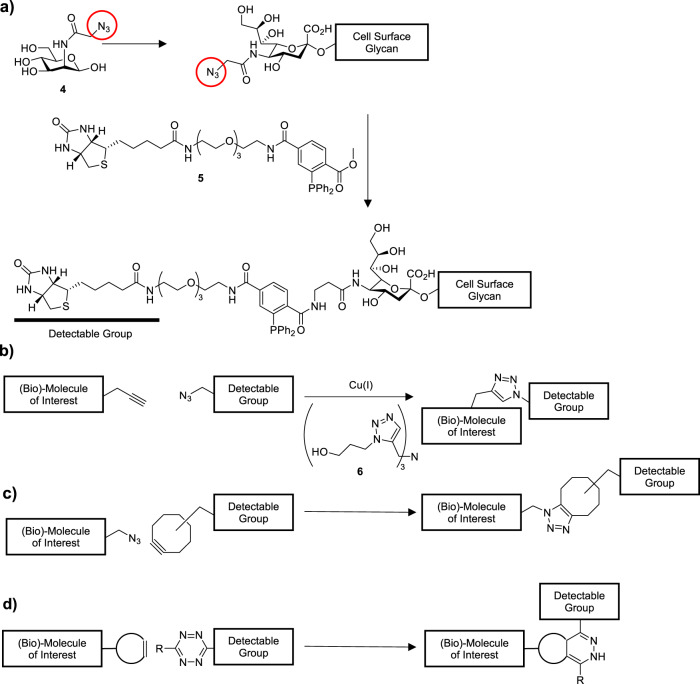
Overview of copper catalyzed and live-cell compatible click reactions. **a** Metabolic glycan labeling: N-acetylmannosamine is modified with an azide group, circled in red, to give N-azidoacetylmannosamine 4. This modification is tolerated by all metabolic enzymes involved in the introduction of sialic acids on glycoproteins and glycolipids on the cell surface. The resulting azido-glycans were subsequently modified with biotin-phosphine reagent 5. This resulted in biotinylation of the sialic acids, allowing their on-surface quantification. **b** The copper-catalyzed click reaction between an alkyne and an azide to yield a 1,4-triazole is one of the lowest background bioorthogonal reactions. Unfortunately, the potential toxicity of the copper catalyst minimizes its use in live cells; **c** This was circumvented by adding ring strain to the alkyne reagent to give the strain-promoted alkyne-azide cycloaddition reaction. Here no catalyst is needed due to the increased reactivity of the alkyne; **d** The inverse electron-demand Diels-Alder reaction is an oft-used live-cell compatible reaction. The strained double bond can react with a tetrazine to form a dihydropyridazine ring between after ligation.

Saxon and Bertozzi [1] reported the first example of what came to be known as bioorthogonal chemistry, an effort that was recently recognized with the 2022 Nobel Prize in Chemistry. The motivation for their work was to quantify changes in a subset of cell surface carbohydrates that contain sialic acid-sugars. These structurally distinct glycans are made as mixtures of *glycoforms* by a set of different enzymes and are difficult to differentiate with antibody-based detection reagents. Their concentration and composition are also not measurable by quantifying changes in gene expression or protein levels. Saxon and Bertozzi’s innovation was to label a precursor of the sialic acid, *N*-Acetylmannosamine (**4**, ManNAz, Fig. 2a) with a small chemical group called an azide (Fig. 2a, circled in red). These azide groups are designated as “bioorthogonal” because they did not interfere with the enzymes that convert ManNAz into azido-sialic acid and into the carbohydrates of cell surface glycoproteins and lipids. Once incorporated into the cell surface carbohydrate of interest, the modified ManNAz was then reacted with a biotin-containing phosphine reagent (**5**, Fig. 2a) which selectively ligated to the azide in a modification of the Staudinger reduction reaction first reported in 1919 [2]. Labeled glycans could then be quantified using flow cytometry or imaged using microscopy [3].

This novel 2-step approach of introducing a biorthogonal substrate followed by selective labeling made it possible to examine and quantify a broad range of substrates and cellular activities. It led to a flurry of development of new reactions and substrates (excellently reviewed in [4, 5]) and became the main application of another nascent chemical field: so-called ‘click chemistry’. This term, dubbed by Sharpless et al. [6] applies to chemical reactions that are sufficiently fast, efficient, solvent-independent and selective that they can be applied in any synthetic strategy. These properties also made them very useful for doing bioorthogonal chemistry, so much so that the terms ‘bioorthogonal chemistry’ and ‘click chemistry’ have become effectively synonymous.

The prototypical click reaction is the *copper-catalyzed [3* + *2] Huisgen cycloaddition reaction* (CCHC, Fig. 2b). CCHC is the reaction between an azide and an alkyne chemical group, catalyzed by copper in its 1+ oxidation state [7]. These two chemical groups are essentially unreactive toward the chemical groups naturally found in cells [8], but upon addition of the copper catalyst, the two groups rapidly react with one another to form a stable triazole product. The CCHC was first used as a bioorthogonal reaction in the chemical modification of a virus [9], but it has since been performed in a broad diversity of species [10–12]. Despite its flexibility, the major downside of the reaction is the toxicity of the copper catalyst. In practice this has meant that the click reaction is used mainly on fixed cells, or with copper(I)-binding ligands that minimized the toxicity of this metal, such as trishydroxypropyltriazolylamine (**6**, THPT, Fig. 2b) and aminoguanine that stabilize the copper(I) in the media (Hong et al. 2010).

To circumvent toxicity, many other bioorthogonal click reactions have been developed that do not require toxic catalysts [5]. The most widely used of these are the *strain-promoted [3* + *2] cycloaddition reaction* (Fig. 2c), and the *inverse-electron demand Diels-Alder reaction* (Fig. 2d) [13, 14]. The expanse of new chemistries developed to work in living cells have been extensively reviewed elsewhere and are beyond the scope of this review [15–17].

Instead, our aim is to focus on the way the above approaches—metabolic labeling of a biomolecule with a small chemical group followed by ligation with a detectable group—have been applied to labelling microbial structures, and then delve into how this can be applied to bacterial ecology and evolution.

### Labeling microbial cell structures using click chemistry

A major advantage of using bioorthogonal two-step labelling to label biomolecules is that the chemical groups you introduce to detect your molecule of interest are very small. The effect of this is that the bioorthogonal molecule behaves very similarly—in a biophysical and biochemical sense—to the parent molecule. The alternative approach—labeling of a molecule of interest with a fluorophore [18]—has a larger effect on the structure and can therefore have an unpredictable impact on the organism or the function that is being studied. Click labeling has been widely applied to bacteria, due to its versatility, the tolerance to biorthogonal compounds of many bacterial enzymes, and the relatively easy synthetic access to many bioorthogonally reactive metabolic precursors. Moreover, the approach has been used to label a wide range of bacterial structures, many of which have been, or can be, applied to microbial ecology. These include specific cell wall carbohydrates, lipids, DNA and the proteome [19]. These will be briefly discussed below before studying examples of their application to microbial communities.

#### Cell wall labeling

The Bertozzi-lab were the first label the bacterial cell wall using a bioorthogonal approach [20]. In their set-up, they fed click-reactive analogues of d-alanine, a component of the bacterial cell wall, to different intracellular pathogens by simply adding it to the growth media. They then used a live-cell compatible click reaction to ligate the click handles with fluorophores that could subsequently be imaged using microscopy. This even worked to selectively image bacteria within infected macrophages, as mammalian cells do not process d-amino acids into their proteome. Interestingly, by using a component of the cell wall, this approach was able to distinguish dividing (producing new cell wall) from quiescent bacteria inside living immune cells [20]. In a particularly exciting extension of this work, Maurelli et al. labeled *Chlamydia trachomatis* with click-reactive analogues of the d-alanine-d-alanine dimer to show, for the first time, that this pathogen produces peptidoglycan during the intracellular stages of its life cycle [21, 22]. Related studies have been carried out in other pathogenic [23] and environmental bacterial species [24, 25] to confirm the presence of peptidoglycan in the cell wall or to delimit regions of cell wall synthesis. Mixed bacterial populations have also been characterized with this reagent. The approach was, for example, used to visualize the in vivo gut microbiome of mice. By first labelling the bacterial species with Alkyne-d-alanine, followed by an ex vivo click reaction with an infrared dye (to allow for deeper tissue imaging) and transfer to a host mouse, the fate of the Gram-positive component of transplanted microbiota could be followed inside the recipient mouse gut [26], thereby showing the ability to track individual species within complex populations.

#### Cell wall carbohydrate labeling

The carbohydrate structures of the cell wall have been a major target for labeling. The peptidoglycan of Gram-positive species contains multiple unique carbohydrates, and is produced by enzymes that have a high functional group tolerance. This has allowed its facile labelling with carbohydrate precursors that carry either a fluorophore, or a bioorthogonal chemical group. For Gram-negative species, carbohydrates in lipopolysaccharide are also an attractive labelling target due to the high carbohydrate content and the shared core structures of these glycolipids. These features have facilitated the introduction of modified sugars into the PG and outer membrane [18] and can be used on mixed microbial communities. For example, an azide-containing analogue of the LPS core sugar 3-Deoxy-d-manno-oct-2-ulosonic acid (**7**, KDO-Az, Supplemental Fig. 1) was used to selectively image the Gram-negative component of the mouse gut microbiome [27]. Previous efforts on labelling the gut microbiome with *N*-azidoacetylgalactosamine (GalNAz) in vivo had run into the issue that host cells were also labelled with this carbohydrate [28]. KDO-Az was not only selective for the bacteria over the host, but also for Gram-negative over Gram-positive species. Chen et al. [27], showed that the dividing Gram-negative commensals could be labelled after ex vivo culture and sorted by FACS and subsequently analyzed by 16S rDNA sequencing. The nature of the labeling again allows for selective labeling of those cells that are constructing cell wall.

Click chemistry is also proving useful for studying the uptake of resources, for example, how commensal species in the gut salvage nutrients from their host by measuring uptake of bioorthogonally tagged host carbohydrates. For example, an azide-containing Fucose (Az-Fuc) was used by the Wu-group to label *B. fragilis* and *Parabacteroides distasonis* [29]. Az-Fuc was taken up via their native fucose salvage pathway and incorporated into their glycoproteins. A similar approach was recently used by Wolan et al. [30], who used a bioorthogonal variant of sialic acid *N*-acetyl-9-azido-9-deoxy-neuraminic acid (Sia9Az) to label the bacterial species in the fecal microbiome capable of salvaging this mammalian carbohydrate. A live cell-compatible click reaction with a fluorophore then allowed the selective labelling of only those bacteria that had incorporated the labelled sialic acid. FACS sorting, followed by 16 rRNA sequencing was then used to identify a new strain of *E. coli* that could incorporate this glycan.

#### Lipid labeling

The lipid bilayer of the bacterial cell wall has been a recent major target of labeling with clickable reagents [18]. The two sphingosine analogues ω-N3-sphingosine and ω-N3-C6-ceramide, were used to study the mechanism of toxicity of these antibacterial lipids during interaction with the cell wall of *N. meningitidis* [31]. The nascent outer membrane of Gram-negative bacteria has also been studied using clickable lipids. For example, 1-propargyl-choline [8] and 1-azidoethyl-choline [9] could be incorporated into phosphatidylcholine analogues **10** and **11** (Supplemental Fig. 1) [32]. This may provide an alternative to the ^13^C labelling of the outer membrane of Gram-negative species in complex microbial communities [33].

#### DNA labeling

Click labeling has been successfully developed to track DNA replication by using a thymadine analogue (5-ethynyl-2′-deoxyuridine) called EdU that is selectively incorporated into replicating DNA [34]. Although EdU-click was developed for use in cell lines or animal models, it has also been applied to complex microbial communities. The appeal of this approach, which strongly complements the BONCAT methods described in more detail below, is that it can be used to distinguish replicating from quiescent cells within mixed communities and then selectively isolate or image these cells for further consideration. For example, Smriga et al. [35] used EdU-click to quantify the frequency of dividing cells in samples of marine bacteria. More recently, EdU-click was combined with FACS-seq to identify the replicating cells within the murine microbiome [36]. Although the results were promising and could, for example, quantify changes in the replicating bacteria within the fecal microbiome before and after antibiotic exposure, it also highlighted potential limitations. The most important limitation is that EdU does not label all bacterial species, including two *Bacteroides* species in the murine microbiome. It remains unclear why EdU is incorporated by some species and not others; however, the difference does not seem to be as simple as a coarse Gram−/+ partition. A second limitation is that incorporation is growth rate dependent, meaning that EdU-click may be less amenable to low resource conditions or situations with slow growth. Overcoming these limitations will considerably broaden the utility of this promising tool.

#### Labeled resources

**Fig. 3:**
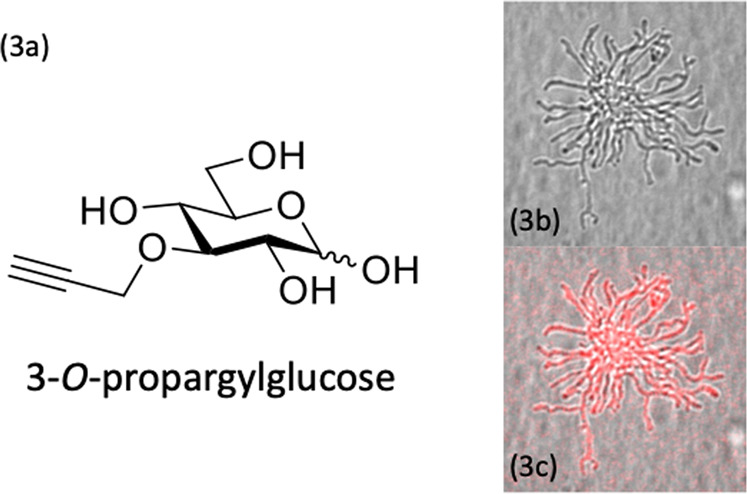
Using click-labeled glucose to track nutrient uptake. The click-labeled glucose analogue *O*-propargylglucose (**a**) can be used as a sole carbon source to support filamentous growth of the multicellular bacterium *Streptomyces coelicolor* (**b**). The image in (**c**) shows the same colony after staining with AlexaFluor647-Azide. All images were taken with a Zeiss Z1 Airyscan with a 40× wet objective (images provided by Niels Cornelissen).

All labeling strategies considered thus far have targeted anabolic pathways in bacteria. However, one advantage of the small chemical groups used in click chemistry is that they are so small that they can also be used to label bacterial resources. One attractive target in this effort is the click-chemistry based tracking of glucose uptake. The fluorescent glucose analogue NBD-Glc (**12**, Fig. S1), has long been used to study Glc-uptake by activated mammalian and bacterial cells [37]. The problem, however, is that the reporter—despite structural similarity to Glc—was not taken up via the canonical glucose transporter in mammalian cells, but instead by an unidentified pathway. In other words, because of the large size of the fluorophore, the chemical properties of the parent molecule are sufficiently altered that its “biological handling” is changed. By contrast, click-chemistry-based *O*-propargylglucose (**13**, Fig. S1) do appear to be taken up via the correct transporter [38]. Our preliminary studies with 2-OPG glucose has already provided evidence for uptake of this analogue in *Streptomyces coelicolor* (Fig. 3). When studying complex populations, these reporters are preferred to prevent skewing of the data by virtue of differential handling of the probe.

### Protein tagging using BONCAT

**Fig. 4:**
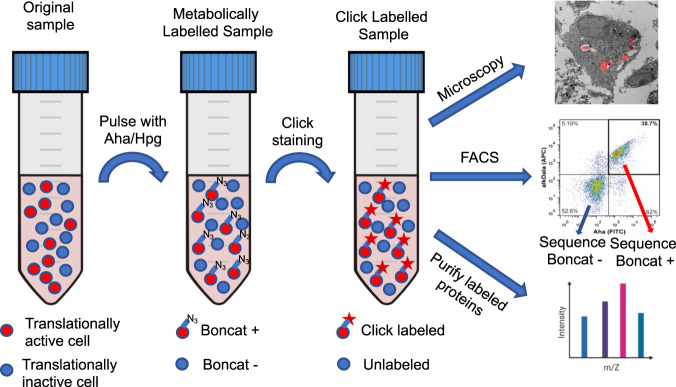
Schematic of a standard BONCAT workflow. In vivo or in vitro samples that vary in translational activity are pulsed with a biorthogonal methionine analog (Aha or Hpg), after which they are labeled for downstream analysis using any type of microscopy, such as the correlative light-electron microscopy of a click-labelled *Salmonella typhimurium* inside a macrophage imaged here [84]; by cell sorting (a mixture of dual cell wall/proteome labeled *M. tuberculosis* and unlabeled cells is shown here) [95]; or mass spectrometry for expressed proteome analysis.

Bioorthogonal non-canonical amino acid tagging (BONCAT) is arguably even more broadly applicable to bacteria and archaea. The idea behind BONCAT is straightforward, leading to a standard workflow (Fig. 4) that has been applied in a range of in vitro and in vivo contexts. Cells are grown in the presence of an amino acid analogue containing an azide tag (red circle in Fig. 2a) that can be subsequently ligated to any of a variety of detectable fluorescent or other labels, depending on the experimental objective. Although different analogues are possible, the most widely used are AHA (azidohomoalanine) [39] and HPG (homopropargylglycine) (Fig. 1) [40]. Both are methionine analogues that are transported into cells via unknown mechanisms and then incorporated into nascent proteins by the promiscuous methionyl tRNA synthases. Virtually all bacterial and archaeal proteins contain at least one Met-residue, thus ensuring the accessibility of the entire proteome to labeling. Neither analogue appears to compromise microbial growth or physiology at low concentrations, and incorporation occurs under a broad range of temperatures and pH, with the exception of very basic pH or high sulfide concentrations. It also works with culturable and non-culturable cells, with minority populations, and with fast or slow-growing microbial populations. Importantly, BONCAT is technically easy to use and combine with other approaches that are already part of the standard microbial ecology and evolution toolkit.

The power of BONCAT comes from its selectivity: only cells that are translationally active will incorporate the amino acid analogue into all proteins produced during exposure to the tagged compound while cells that are quiescent will remain unlabeled (Fig. 4). This difference makes it possible to partition complex microbial communities into active and inactive fractions which can then be visualized in situ using fluorescence microscopy or separated using cell sorting (FACS) for downstream analysis by any number of established -omics approaches. Alternatively, because AHA and HPG are specifically incorporated into actively translated proteins, BONCAT-labeled proteomes provide a quantitative assessment of which proteins are enriched or differentially regulated in a given condition [11]. In this approach, BONCAT-labeled proteins are affinity purified and then subjected to mass spectrometry. As with BONCAT-FACS that isolates only functionally active species from a mixed population, BONCAT proteomics only labels actively translated proteins, which can be used to identify pathways that are induced under certain conditions. Several groups have used this method to analyze mechanisms of pathogenesis and virulence [41], quorum sensing [42], and the response to antibiotics [43]. Identifying proteins associated with a given environmental shift does not provide direct causal evidence of their functional importance; however, it can help to focus attention on important pathways that can be further examined using targeted knock-outs or overexpression.

### BONCAT in natural/industrial populations

Hatzenpichler et al. [44] and a contemporaneous study from Samo et al. [45] provided the first evidence of the breadth and potential of BONCAT in natural systems. After confirming the suitability of the MET analogues AHA or HPG to label translationally active cells after short incubations in pure cultures, they tested the applicability of the approach in complex microbial communities using the basic workflow shown in Fig. 4. These, and a later companion study [46], led to several key insights: (1) that BONCAT could label physiologically and phylogenetically diverse microbial groups; (2) that it relied on protein synthesis, rather than growth per se, and so could label very slowly or non-dividing cells; (3) that it could distinguish translationally active from inactive cells in diverse communities; (4) that analogues could be applied in situ, for example to label cells in oral biofilms or sediments; (5) that it labeled cells very quickly, i.e., within the generation time of cells; and (6) that it could be easily combined with other established methods, such as FISH, FACS and sequencing, to identify which cells and species were active at any given point in time. BONCAT-FISH combines the standard workflow (Fig. 4) with taxon-specific hybridization probes to label specific groups of bacteria while simultaneously assessing their translational activity. BONCAT-FACs can sort active from inactive cells to quantify their frequencies or for further characterization using sequencing or proteomics.

Several groups have used this experimental framework to address fundamental questions in microbial ecology in natural, industrial, and host-associated environments. Couradeau et al. [47] used BONCAT to characterize the translationally active fraction of soil bacteria from two different soil depths: 30 and 76 cm. They found that some 20 and 60% of bacteria were active at the two depths, respectively, far more than had been estimated in other studies, possibly because BONCAT is not reliant on bacterial division. Next, using FACS followed by sequencing to isolate the BONCAT+ cells from each location, they found that the active microbial fraction was a taxonomic subset of the unlabeled cells, showing that only some species are active at any given time, and also that the dominant taxa varied at the different soil depths. Similar results have been obtained from marine samples [48, 49], anammox consortia [50], coal mines [51], and activated sludge [52]. Results to understand in vivo microbiomes using BONCAT remain limited, but also have considerable potential to provide unique insights into these complex communities. Valentini et al. [53] used BONCAT-FACS to examine the active fraction of bacteria in the sputum of CF patients, and Taguer et al. [54] to study the gut microbiota. While both studies determined that only a fraction of cells were translationally active at any given time, they found fewer taxonomic differences between the BONCAT+ and the bulk communities than in environmental samples. It remains to be seen if this is reflective of other host-associated communities.

In each of these examples, BONCAT made it possible to amplify the experimental signal from functionally important species by focusing exclusively on active cells, however rare and regardless of growth or growth rate, while ignoring (or putting aside) the potentially greater signal from either dormant cells, dead cells or eDNA.

## Labeling of bacterial enzymatic activities with click chemistry

One final approach in which bioorthogonal labels can offer important insights into bacterial physiology is through its use in labeling specific enzymatic activities in bacteria [55]. This was, for example achieved by labelling carbohydrate precursors and the metabolic incorporation into peptidoglycan [56, 57], bacterial lipopolysaccharides [56], or even bacterial protein glycosylation [58]. Over the last few decades, the field of activity-based protein profiling has also made great strides. By using covalent inhibitors of specific enzymes linked to a fluorophore [59], specific enzyme activities could be visualized for individual bacteria. As with previous examples, a common problem when studying bacterial enzymes in this manner is the change in biophysical properties of the pendant fluorophore. This too is circumvented using click chemistry. In the earliest example, Speers and Cravatt labelled a sulfonate ester with an azide group and visualized serine hydrolase activities in cells using a copper-catalyzed click chemistry approach [60]. This approach was rapidly translated to the study of bacterial enzyme activities [61], such as bacterial glycosidases [62] and transferases, as well as the enzymes involved in host-pathogen interaction [63]. A recent example of this work that highlights a future direction is the combination of enzyme activity labelling with fluorescence in situ hybridization. Using this approach, Sakoula et al. [64] could identify new ammonia and alkane oxidizing bacteria from complex populations.

## Potential applications of click chemistry and BONCAT

There is broad potential to integrate click-labeled biomolecules into ecological and evolutionary applications. They are relatively easy to use, are less expensive than related methods, and can be easily combined with other established tools. Our aim below is to outline several possible applications; this is by no means meant to be exhaustive and only reflects our own current interests.

1. Natural or synthetic communities: Different species/strains in mixed communities may have temporally or spatially distinct or overlapping responses to stress or resources. BONCAT or click labeling with d-Ala analogues [20] can be used to isolate translationally active cells and bacterial cell walls after pulsing with a stressor, or a natural or anthropogenic substrate, and then examined via mass spectrometry proteomics to identify the pathways that are induced in response. This could rapidly identify novel mechanisms associated with toxin or pollutant degradation and could help to partition the roles of different taxonomic groups by exposing communities to single substrates in series. Assimilation of complex substrates via cooperative degradation or cross-feeding could be similarly examined, as could transport and metabolism of click-labeled resources (Fig. 3).
2. Microbiomes in situ: Plant and animal microbiomes are responsible for a broad range of essential functions for their multicellular hosts [65, 66]. BONCAT has been used to study microbial responses in CF lungs and the gut microbiota. These can be expanded by tracking community dynamics during host exposure to different resources, stresses or pathogens, and followed by the approaches noted above. Spatial mapping of biofilms on roots, tissues, or complex substrates can be measured with FISH and integrated with BONCAT to determine activity through time and space. A key advantage is the ability to rapidly separate active from inactive cells via FACS, making it possible to identify functionally important microbes down to the strain level while simultaneously measuring protein production.
3. Experimental evolution: Experimental evolution tracks the evolution of bacterial strains or communities during serial passage over 10 s–1000 s of generations [67, 68]. Especially in the context of complex communities, BONCAT proteomics can help to partition communities into functionally active or inactive species during exposure to mixed substrates, to track community shifts during evolution of synthetic consortia or to identify functionally important proteins or pathways that are enriched during exposure to stress or complex resources [69, 70]. In more classical experimental evolution approaches with single species, BONCAT can also potentially complement sequencing studies used to identify newly fixed mutations with quantitative analyses of altered protein expression.
4. Population heterogeneity in single or multicellular microbes: It is increasingly clear that bacterial clones can express significant phenotypic heterogeneity due to gene expression noise or other sources of instability [71]. Persisters or heteroresistant cells within isogenic bacterial populations can survive antibiotic exposure while the remainder of the population is killed [72]. Similar heterogeneity affects the duration of lag phase [73], transitions to new resources during diauxic shifts [74], production of antibiotics or bacteriocins [75, 76], and many others. Click-based approaches can be used in these cases to isolate rare subpopulations that are translationally active (BONCAT), replicating (EdU) or actively transporting and growing on click-labeled carbon sources. Multicellular bacteria and fungi also show spatially heterogeneous behavior in terms of growth, secretion, transport and response to exogenous conditions [77, 78]. BONCAT and other click-labeled compounds can be combined with detailed microscopy to identify translationally active regions of multicellular colonies, including filamentous microbes, as well as the intercellular translocation of labeled substrates. 2-NBD-Glc and a fluorescent sucrose analog, esculin, have been used for this purpose in filamentous fungi [79] and cyanobacterial filaments [80], respectively; click-labeled compounds whose transport and metabolism is less compromised by pendant fluorophores may produce results that are closer to what occurs with native substrates.
5. Other microbial groups and ecological interactions: BONCAT has not yet been as widely applied to the ecology and evolution of non-bacterial microbes. Two studies, one in bacteria [81] and another in a marine protist flagellate [82], showed that BONCAT could label new phage and viral proteins after pulsing their hosts with HPG or AHA, respectively. This made it possible to quantify viral replication by tracking the production of new viral particles using high-resolution microscopy. By the same approaches as above, BONCAT could also be used to quantify the defensive proteome of cells when they are targeted with biotic enemies, or to decipher mechanisms of mutualistic interactions between species.

## Limitations of click chemistry and BONCAT

Despite major advances in the field of labelling non-templated biomolecules, click-chemistry-based approaches are not without their limitations. The first of these is the toxicity of some of the labels that can reduce growth rates, viability or change cellular metabolism. For example, although neither HPG nor AHA had any detectable impact on *E. coli* growth, both caused measurable shifts in a small fraction of metabolites, especially when cells were exposed to analogues during heat stress [83]. By contrast, using HPG as a methionine analogue in *Salmonella typhimurium* [84] and *Mycobacterium tuberculosis* decreased growth rates when the labels were given over longer time periods. Similarly, HPG in marine *Synechococcus* reduced growth at high concentrations while also affecting protein stress and energy production [85]. Although these effects were largely mitigated at lower doses and by modifying the pulse length with the label (as a rule of thumb a maximum of two cell division times is used), this potential limitation needs to be considered in different species in different environmental contexts.

A second type of toxicity stems from some of the reagents used for some of the bioorthogonal ligation reactions. One of the most-used ligation reactions, the copper-catalyzed Huisgen ligation makes use of a Cu(I) catalyst, which is toxic to most cells. Applications of this reaction are therefore limited to those where the bacteria are either fixed before labeling, or analyzed very shortly after live-cell labeling. The use of stabilizing ligands for the Cu(I) can also minimize the toxicity [86]. The availability of other live-cell compatible click reactions, such as the strain-promoted click-reaction and the inverse electron-demand Diels-Alder ligation reactions, and of fixed-cell compatible sequencing approaches have in part negated this problem [15].

Bioorthogonal chemistry is a field that is still in development. Many bioorthogonal ligation reactions are still not completely bioorthogonal. In many cases, the reagents used are not stable over long periods of time in biological samples. For example, the cyclooctynes used in the strain-promoted [3 + 2] cycloaddition reaction can react with thiols present in biological samples [87], or can be destroyed by other biological mechanisms, such as the oxidative burst that occurs in the macrophage phagosome [8]. This means that great care must be taken selecting the correct reaction for the system that one wishes to study. Good guidelines have been reported for this by Prescher et al. [88].

Differential label incorporation may be problematic in multi-species communities. This can occur if some species are intrinsically less efficient at incorporating the label of choice or if there is competition for uptake with the unlabeled biomolecule. For example, EdU is unable to label all bacterial species [36], for reasons that remain unclear. In addition, both AHA and HPG are incorporated less efficiently in the presence of methionine [44], which may be a limitation in environmental or host-associated environments containing exogenous methionine. Because there is no way of checking this a priori when using these approaches it is worthwhile to assess label incorporation at different concentrations and times, to assess the variation in species detection. In addition, development of new biorthogonal amino-acid analogues, such as the clickable threonine analogues recently reported by Bonger et al. [89] can expand the range of conditions that are suitable for cell labeling.

## Summary

No single set of methods can answer the huge breadth of questions occupying the time and attention of microbial ecologists. This is equally true for the click-based approaches we have introduced above. However, despite some limitations, we believe that metabolic probes that target different microbial biomolecules provide a powerful means to study multi-species bacterial populations in complex environments. A click-chemistry-based strategy can rapidly and inexpensively identify species or strains that are metabolically active and can be readily integrated into existing multi-omics pipelines. Looking forward, we are particularly excited by rapid developments in the field from the chemical side, such as the combination of click chemistry with activity-based protein profiling [90], where the activity of individual bacterial enzymes can be studied and profiled. A second development is click-multiplexing, where multiple bioorthogonal reactions are combined to study different metabolites in a single sample [91–95]. One of us (SvK) had used this, for example, to study the fate of both the PG-layer and the proteome of *M. tuberculosis* growing within a macrophage [95]. These developments, as well as the continuing emergence of new bioorthogonal ligation reactions and labeled resources, promises that the potential for click-chemisty approaches in microbial ecology and evolution will continue to expand.

## Supplementary Information

Figure S1
