# Supplementary figures and images for: Using click chemistry to study microbial ecology and evolution
Source: ISME Commun. 2023 Jan 31;3:9. doi: 10.1038/s43705-022-00205-5 (PMC9889756; doi:10.1038/s43705-022-00205-5)

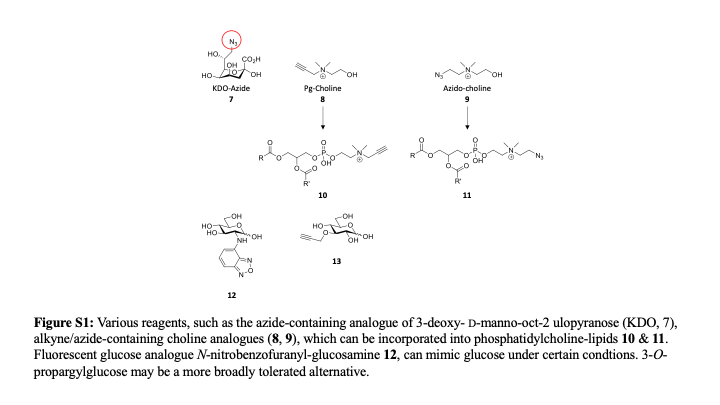

Supplement: Supplementary file 1 — Figure S1 [file 43705_2022_205_MOESM1_ESM.tif]
